# Evaluation for the optimization of two conceptual 200,000 m^3^/day capacity RO desalination plant with different intake seawater of Oman Sea and Caspian Sea

**DOI:** 10.1007/s13201-020-01338-5

**Published:** 2021-01-14

**Authors:** Fatemeh Yusefi, Mir Mahdi Zahedi, Morteza Ziyaadini

**Affiliations:** Department of Marine Chemistry, Faculty of Marine Sciences, Chabahar Maritime University, P.O. 99717-56499, Chabahar, Iran

**Keywords:** Optimization, Evaluation, Reverse osmosis desalination, Oman sea, Caspian sea

## Abstract

Iran has faced with water scarcity problem for a long time. There is a strong tendency to desalinate seawater from Oman or the Caspian Sea as intake seawater and transfer it to central parts of the country. These projects face significant technical, economic, and environmental challenges. In this work, utilizing available economic theories about single-stage reverse osmosis (RO) desalination plants, the cost analysis of a conceptual plant with a production capacity of 200,000 m^3^/day, was accomplished assuming the use of Oman and Caspian seawater as feed. The effect of important parameters such as applied pressure, recovery ratio, total salt content of the feed, and produced water and the temperature has been studied theoretically. The results show that under the same working conditions, the final product price per cubic meter of freshwater from the Caspian Sea is $ 0.69 versus $ 1.24 for the Oman Sea, which is about 50% cheaper. The lower salinity of the Caspian Sea compared to the Oman Sea is the main reason, which lead to reduce in the capital cost of the RO membrane (62% difference), cost of the intake and pretreatment (20%), and cost of membrane elements replacement (13%) regardless of water transfer cost.

## Introduction

Today, with an increasing population and more and more freshwater consumption, climate change, and uneven rainfall distribution, we face sharp increase in freshwater scarcity all of the world (Oki and Kanae 2006; Debele 2019; El-Emam and Dincer 2014). Lack of rainfall has detrimental effects on industrial, agricultural, domestic, and ecological activities. Historically, Iran has been faced with the water scarcity due to its dry climate, especially in the central and east west area of the country (Farhoudi and Poll 1992, Daneshmand and Mahnoudi 2017). According to the report of the Parliament Research Center of IRAN, in the last ten years, the amount of rainfall has decreased by about 11% compared to the long-term average. Due to droughts and climate change, the total amount of renewable water from 130 BCM has decreased to 89 BCM. However, the total amount of consumed water is 96.37 BCM (https://rc.majlis.ir/fa/news/show/1040385). The recent outbreak of the new coronavirus (COVID-2019) has led to an increase in water consumption to reduce the spread of the disease.

To overcome the freshwater scarcity, require a new source of potable water that can be supplied by seawater desalination methods (Van der Bruggen and Vandecasteele 2002, Wilf and Schierach 2001). The global desalination industry rapidly has developed the thermal and membrane-based seawater desalination process. In the meantime, the use of multi-stage flash (MSF), multi-effect distillation (MED), and especially reverse osmosis (RO) processes has been considered, due to improvement of their reliability and the performance of freshwater production (Elimelech and Phillip 2011). Rapid growth in membrane technology (Yang et al. 2018) is primarily based on the correct understanding of the potential of this technology. RO is now becoming a leading technology for brackish and seawater desalination (Wenten and Khoiruddin 2016). A reverse osmosis (RO) system uses a semi-permeable membrane to remove ions, proteins, and organic chemicals, which are generally not easily removed using other conventional treatments (Sarai Atab et al. 2016). Scientists try to reduce these limitations of RO desalination, including the development of novel membranes with high water and low salt permeability, energy consumption and fouling, and either to improve the various suitable techniques for intake water pretreatment and produced water post-treatment (Voutchkov 2018, Zahedi and Ghasemi 2017, Pourmortazavi et al. 2017).

It is essential that the project planners and desalination engineers provide cost analysis regularly for this business cases (Al-Obaidi et al. 2019, La Cerva et al. 2019). The cost of water production includes all spending associated with project implementation consists of fixed and variable components. The fixed water costs are payments for plant construction and the capital investment in the plant (i.e., capital cost recovery). Also, it encompasses the part of the annual O&M spending that are independent of the actual volume of water produced by the desalination plant (labor, maintenance, environmental, performance monitoring, and indirect O&M costs). The variable cost of water incorporates O&M expenditures that are directly related to the actual volume of produced desalinated water (power, chemicals, replacement of membranes and cartridge filters, and waste stream disposal) (Voutchkov 2013, Lee et al. 2011). The economics of SWRO and the cost of the product water are of great importance and affect the choice of the design and operating parameters (Malek et al. 1996, Marcovecchio et al. 2005).

**Fig.1:**
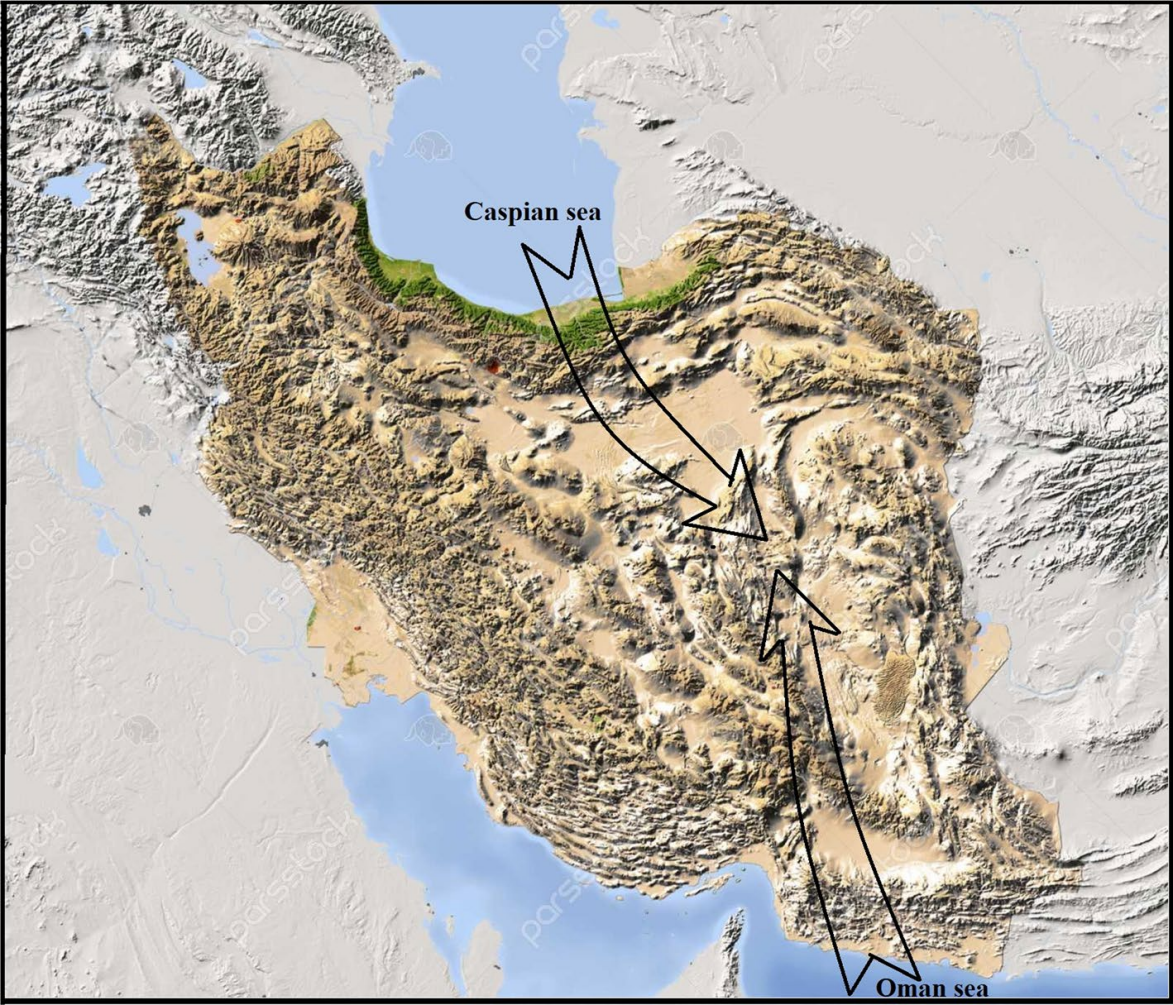
Topographic map of Iran and two proposed ways to transfer desalinated seawater to its central regions

As mentioned earlier, Iran has also faced with freshwater scarcity problem for a long time, and there is a strong tendency to desalinate seawater from Oman or the Caspian Sea and transfer it to central parts of the country (Fig. 1). Here, in this study based on our last published article (Emamjome et al. 2019) the theoretical modeling was modified for calculation of cost-effective part of two conceptual RO desalination plant with the capacity of 200,000 m^3^/day with different intake seawater of Oman Sea and Caspian Sea such as capital cost of the RO membrane, cost of the intake and pretreatment, and cost of membrane elements replacement.

## The conceptual SWRO system and economic analysis algorithm

**Fig.2:**
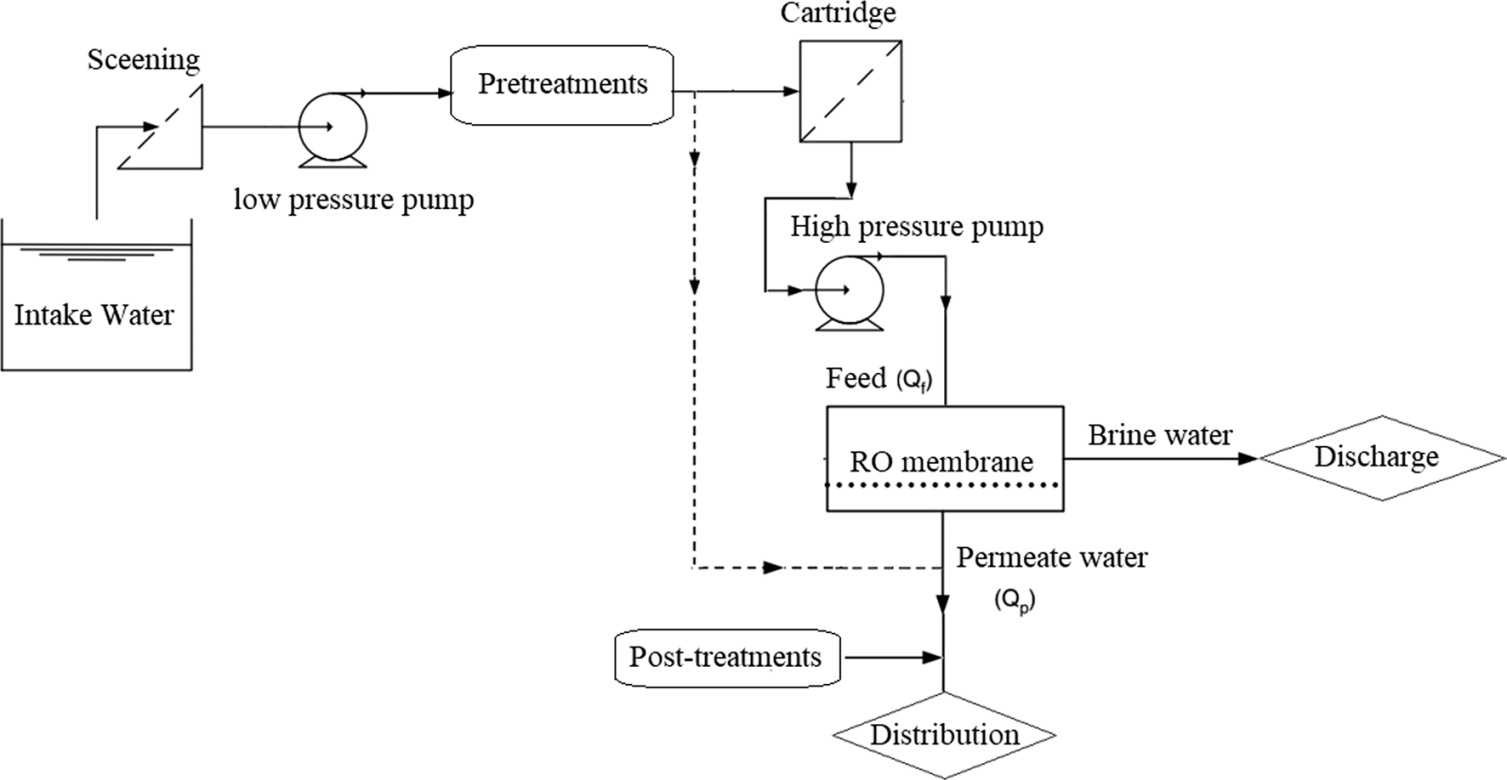
RO desalination system

The SWRO concept unit proposed in this study produces 200,000 m^3^/day, while the salinity of the water produced is under 500 ppm. In this working system, the seawater is pumped into the system by a low-pressure pump (Fig. 2). After passing the pretreatment process via high-pressure pump are injected into RO membrane series. Part of the feed passes through the membrane related to the recovery ratio (*r*_*r*_) as a permeable product and enters the distribution network after subsequent post-treatment processes. On the other hand, the other part of the water, which has a higher salinity, is prepared for disposal.

**Fig.3:**
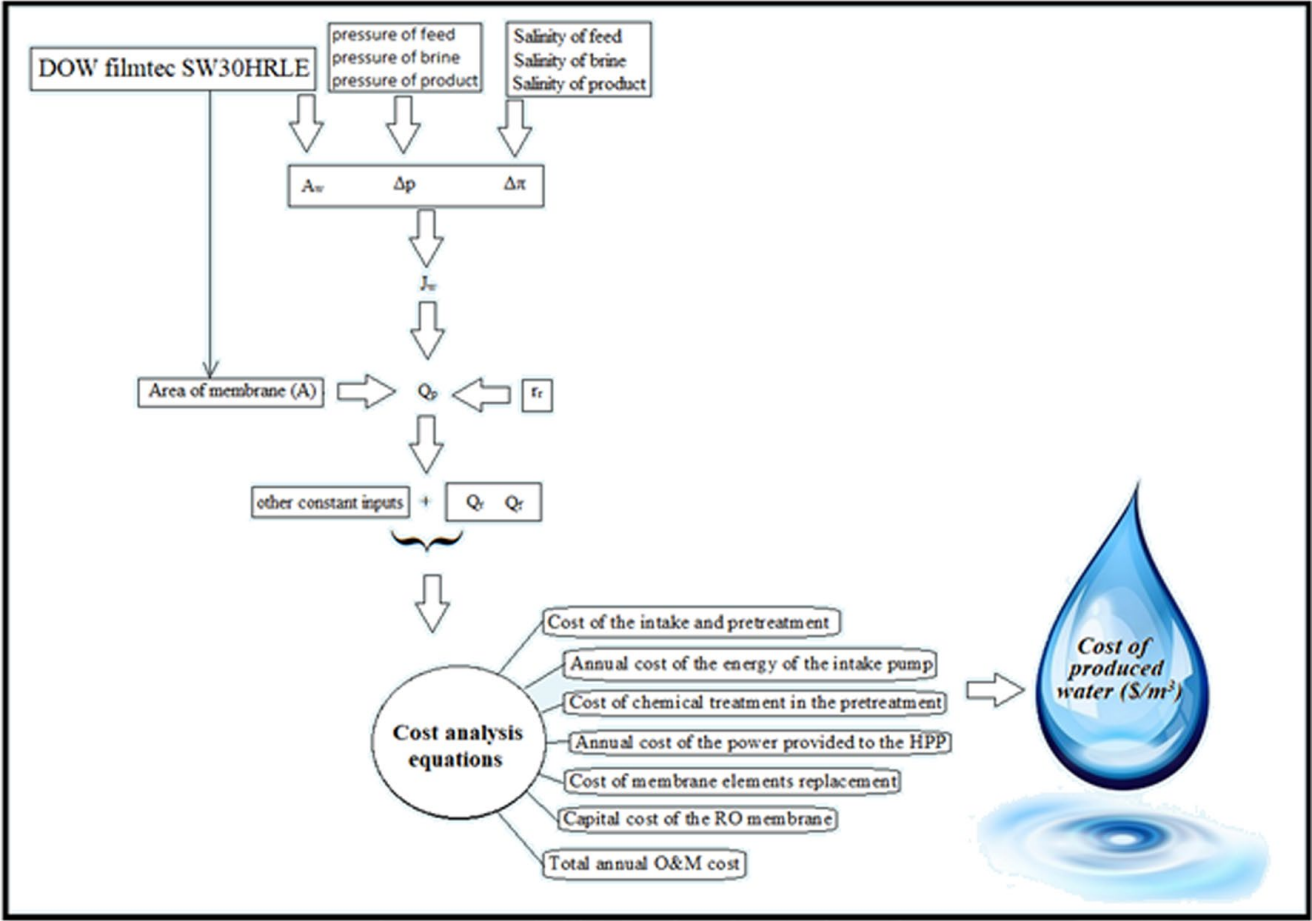
Calculating algorithm for comparative cost analysis

To calculate and compare the cost of desalination, water desalination plant designed with two feedings of the Caspian Sea and the Sea of Oman. The membrane used for both plants was DOW filmtec SW30HRLE, and according to its datasheet, the active area (*A*_*w*_) was used in this work for cost analysis purpose. As we can be seen in Fig. 3, the computational algorithm is calculated by summing the salinity values for feed, saline, and brine, and then osmotic pressure, and static pressure using related formulas (Table 1). As a result, the flow rate (*J*_*w*_) for each membrane could be calculated and then *Q*_*P*_ parameter could be obtained using *r*_*r*_. This parameter (*r*_*r*_) use for calculating the *Q*_*f*_ and *Q*_*r*_ as feed and brine flow rate. These data used as input of cost analysis, the output of this analysis will provide the costs associated with each RO plant with the difference salinity of intake water. Other parameters related to the economic calculations for each of the waters of the Caspian Sea and Oman are given in Table 2 separately by water source. The salinity of feed water in comparative conditions for Caspian and Oman seawater takes 17,000 and 36,000, respectively, but as will see, the effect of this variable on the cost and permeate flow rate is studied.

**Table 1:** Equations of calculation of the capital and operating cost

Description	Equation (Sarai Atab et al. 2016, Emamjome et al. 2019)
Cost of the intake and pretreatment	$\dot{C}_{{{\text{BWIP}}}} = 996\left( {Q_{f} } \right)^{0.8}$
Annual cost of the energy of the intake pump	$\dot{C}_{{e \cdot {\text{BWIP}}}} = \frac{{P_{IP} Q_{f} }}{{\eta_{IP} }}C_{e} f_{1}$
Cost of chemical treatment in the pretreatment	$\dot{C}_{{e \cdot {\text{op}} \cdot {\text{ch}}}} = Q_{f} C_{{{\text{ch}}}} f_{1}$
Annual cost of the power provided to the HPP	$\dot{C}_{{e \cdot {\text{HPP}}}} = P_{{{\text{HPP}}}} \dot{Q}_{f} C_{e} f_{1} /\eta_{{{\text{HPP}}}}$
Cost of membrane elements replacement	$\dot{C}_{{{\text{RO}}}} = NP_{m} CD_{m}$
Average salinity through the membrane element	$\overline{C} = \frac{{Q_{f} C_{f} + Q_{r} C_{r} }}{{Q_{f} + Q_{r} }}$
Area	$A = \dot{Q}_{P} \frac{{C_{{{\text{RO}}}} }}{{B_{s} \left( {\overline{C} - C_{{{\text{RO}}}} } \right)}}$
Cost per membrane	$PC_{m} = 10A$
No. of elements	$N = r_{r} \left( {\frac{{\dot{Q}_{f} }}{{Q_{{{\text{p}} \cdot {\text{el}}}} }}} \right)$
Capital cost of the RO membrane	$PC_{RO} = N PC_{m}$
Total annual O&M cost	$\dot{C}_{{{\text{O}}\& {\text{M}}}} = 0.126f_{1} \dot{Q}_{{{\text{p}} \cdot {\text{a}}}}$
Constant escalation levelization factor	${\text{CELF}} = {\text{CRF}}\frac{{K\left( {1 - K^{n} } \right)}}{1 - K}$
Constant factor	$K = \frac{{1 + r_{n} }}{{1 + i_{{{\text{eff}}}} }}$
Capital recovery factor	${\text{CRF}} = i_{{{\text{eff}}}} \frac{{\left( {1 + i_{{{\text{eff}}}} } \right)^{n} }}{{\left( {1 + i_{{{\text{eff}}}} } \right)^{n} - 1}}$
Osmotic pressure (bar)	$\pi = \frac{{0.0385\left( {{\text{ppm}}} \right)T}}{{14.5\left( {1000 - \frac{{{\text{ppm}}}}{1000}} \right)}}$
Delta osmotic pressure (bar)	$\Delta \pi = 0.5\left( {\pi_{f} + \pi_{b} } \right) - \pi_{p}$
Delta pressure (bar)	$\Delta p = \frac{{P_{f} + P_{r} }}{2} - P_{p}$
Net driving pressure (bar)	${\text{NDP}} = P_{f } - \left( {\Delta \pi + P_{p} + 0.5P_{d} } \right)$
Permeate flux (L/m^2^.h)	$J_{w} = A_{w} \left( {\Delta P - \Delta \pi } \right)$

**Table 2:** Parameters of conceptual RO desalination plant with capacity of 200,000 m^3^/day for two different intake water of Caspian and Oman sea (using membrane element DOW filmtec SW30HRLE)

Operational, flow, and technical parameters	Caspian sea	Oman sea
No. of elements	12,400	78,787
Delta osmotic pressure (bar)	21.7	39.7
Net driving pressure- NDP (bar)	21.2	3.34
Permeate flux (*J*_*w*_—L/m^2^.h)	16	2.54
Feed water flow rate m^3^/day	363,000	500,000
Salinity of feed water (ppm)	36,000	17,000
Salinity of product water (ppm)	400	400
Membrane recovery ratio, *r*_*r*_	0.55	0.4
Seawater feeding temperature (°C)	25	25
Pressure of feed (bar)	45	45
High pressure pump efficiency, *η*_HPP_%	90	90
Low pressure pump efficiency, *η*_LPP_ %	90	90
Plant load factor, *f*_1_%	90	90
Membrane replacement factor, *r*_*m*_ %	10	10
Membrane salt rejection ratio %	98.7	98.7
Cost of chemical treatment, *C*_ch_ ($/m^3^)	0.0197	0.0197
Cost of cartridge filters replacement ($/m^3^)	0.01	0.01
Interest rate%	8	8
Nominal escalation rate, *r*_*n*_%	5	5
Economic life time, year	20	20
Effective discount rate, *i*_eff_ %	8	8

## Results and discussion

### Evaluating the effect of applied pressure on the water cost and the permeate flow rate

**Fig.4:**
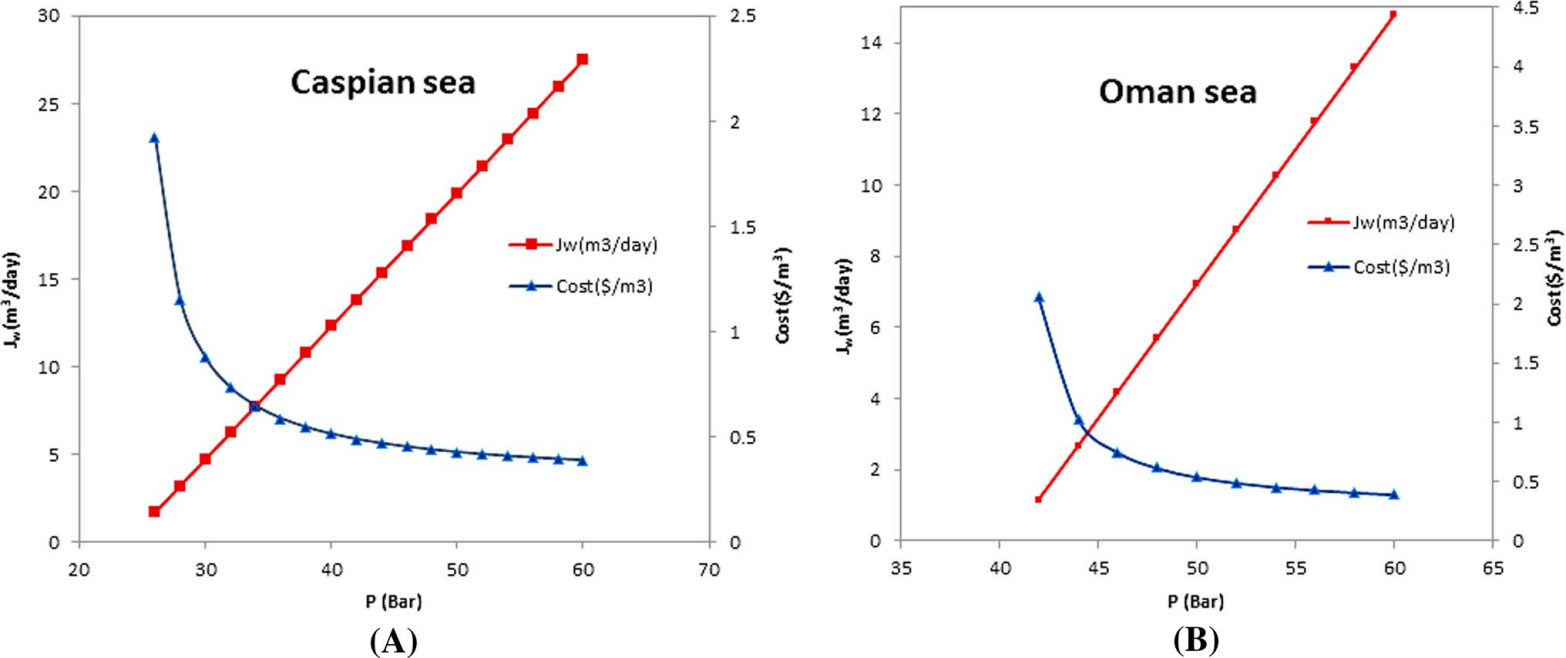
Evaluating the applied pressure on the cost of product water and the permeate flow rate from the membrane

Input feed water of the Caspian and Oman Sea was used to determine the effectiveness of the modeling parameters of the desalination process by the RO desalination method. In this regard, the value of some parameters was measured in a working range and the effectiveness of these RO parameters was investigated and analyzed. Due to the importance of the applied pressure as well as the flow rate (flux) of the membrane permeate water in the reverse osmosis process, changes of these two parameters via the cost of product water per cubic meter have been studied simultaneously in a working range of 26–60 bar for Caspian and 42–60 bar for Oman Sea. The lower limit of this range of applied pressure has been chosen, due to the proportionality with the different salinity of the intakes water and having the least membrane permeate water. You can see the result of this study in Fig. 4a and b for the intake water of the Caspian and Oman Sea. The pressure changes are significant effect on the cost of produce water, and the rate at which water flows through the membrane. Although the selected compression range is different, in both types of input water sources, the change of studied parameters is similar (one linear and the other exponential). The observed changes are classic for both water sources. However, it is clear that under similar pressure conditions, the cost of product water with using Caspian Sea seawater is lower than in the Oman Sea and the flow rate of membrane permeate water is higher. This is due to the direct effect of feed salinity on osmotic pressure.

### Evaluating the effect of recovery ratio on the water cost and the permeate flow rate

**Fig.5:**
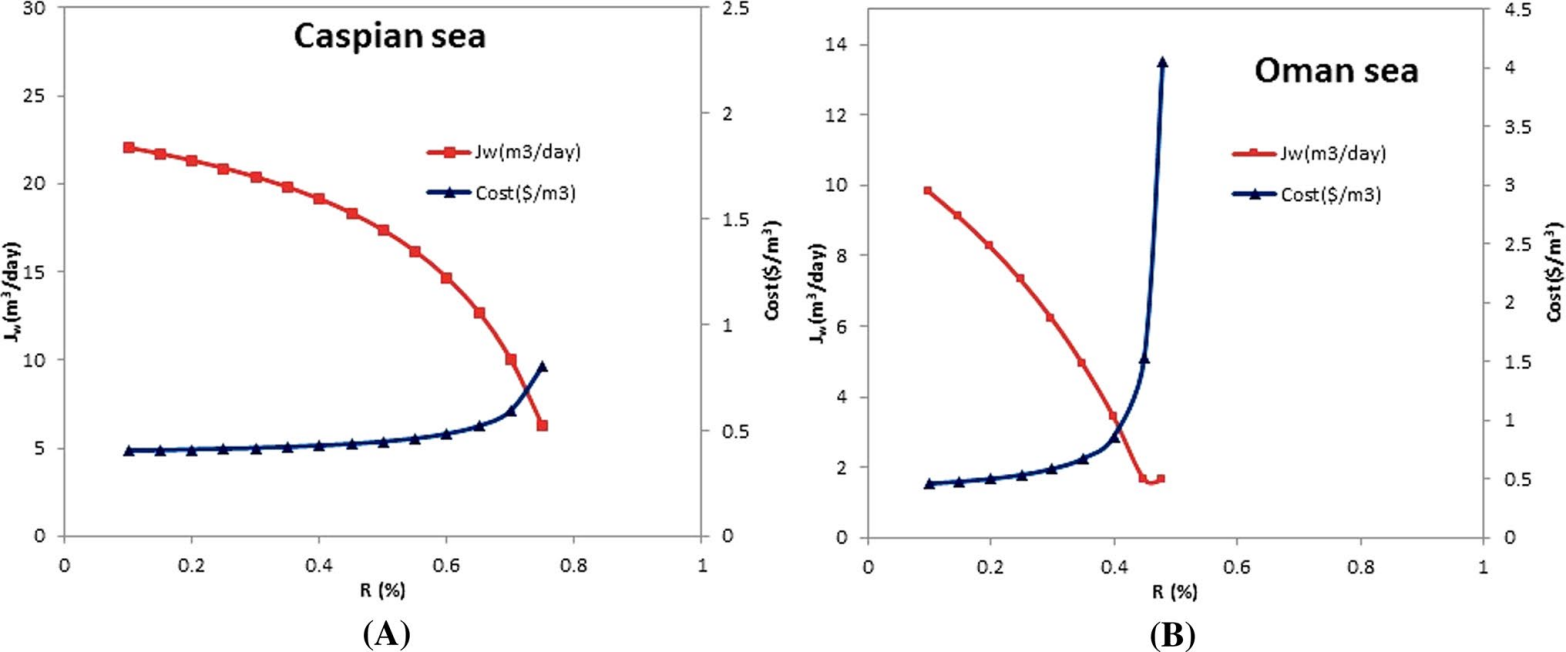
Evaluating the effect of the recovery ratio on the cost and the permeate flow rate (Δ*P* = 45 bar, *T* = 25 °C)

Determining the recovery ratio correctively is another important factor affecting the quality and operations of the reverse osmosis membrane, and on the RO desalination process. The results of the study of the influence of permeate flow rate and the cost of product water with the change of the recovery ratio for the two types of intake water are presented in Fig. 5a and b. The study range for the Caspian Sea and Oman Sea is 0.1–0.75 and 0.1–0.48, respectively. In the case of the Caspian Sea, as can see, changes of recovery affect the permeate water and the cost of product water, so that the rate of permeate of the water decreases significantly with increasing recovery. At the same time, the cost does not change much until the recovery ratio of 0.6, and the cost initially increases linearly and finally exponentially. So the choice of recovery ratio should be such that we have a higher permeate flow. On the other hand, at high recovery rates, the risk of membrane fulling will increase. Regarding the Oman Sea intake water, the changes in the recovery ratio on cost are very significant. As is clear, changes in both parameters with the recovery ratio are nonlinear in both feed cases.

### Evaluating the effect of feed salinity on water cost and the permeate flow rate

**Fig.6:**
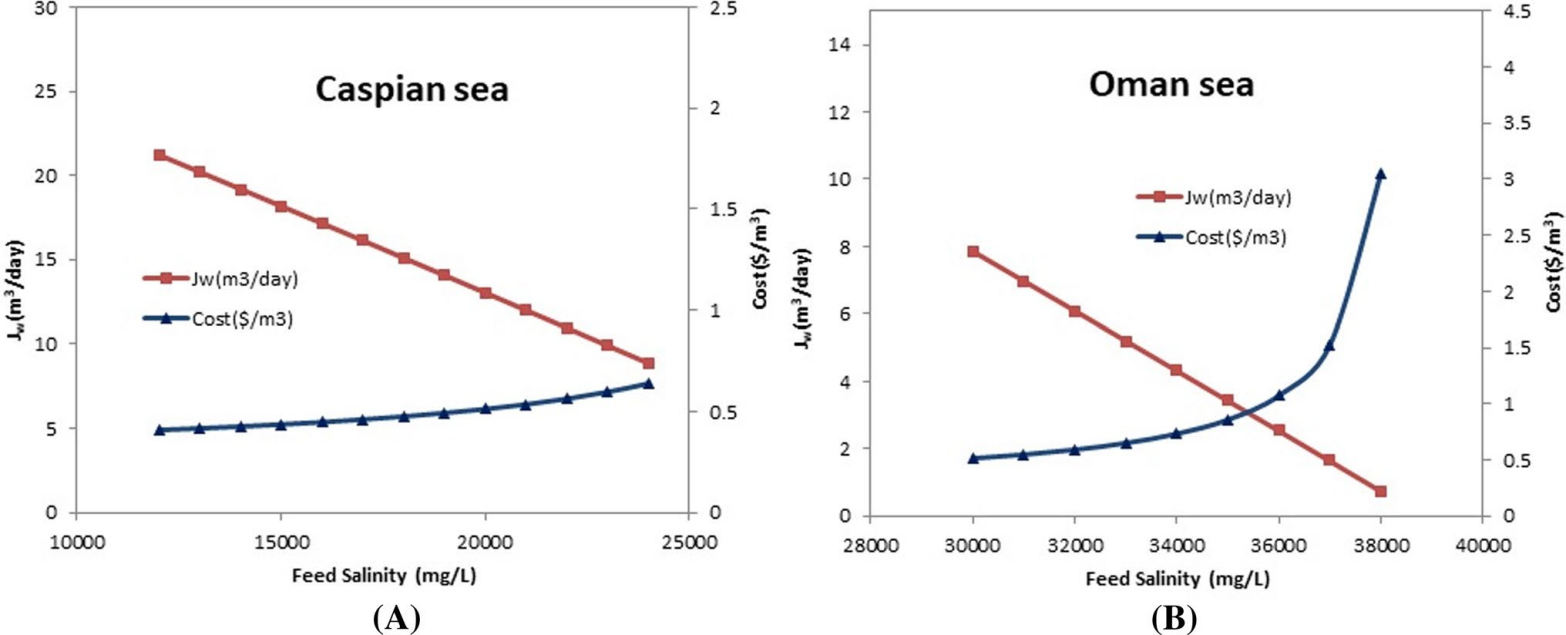
Evaluating the effect of the feed water salinity on the cost and the permeate flow rate (Δ*P* = 45 bar, *T* = 25 °C, *R*_Caspian_ = 0.55, *R*_Oman_ = 0.4)

The salinity of feed water is an important parameter in RO desalination and either the cost of producing parameter freshwater depends on them. Typically, the higher salinity of the feed water entering the water desalination plant led to the higher cost of product water. Therefore, to evaluate the effect of salinity on the cost and permeate flow rate of possible salinity range of intake waters was examined, and the results are shown in Fig. 6a and b. Given the results of cost modeling, it is clear that the range of salinity changes in the input sample has a significant impact on the cost of product water. In the case of Oman Sea feed water, with a salinity of 38,000, the price of product water is received to more than $ 3. The behavior of the permeate flow rate in both cases was similar but with different intercept.

### Evaluating the effect of product water salinity on the water cost and the permeate flow rate

**Fig.7:**
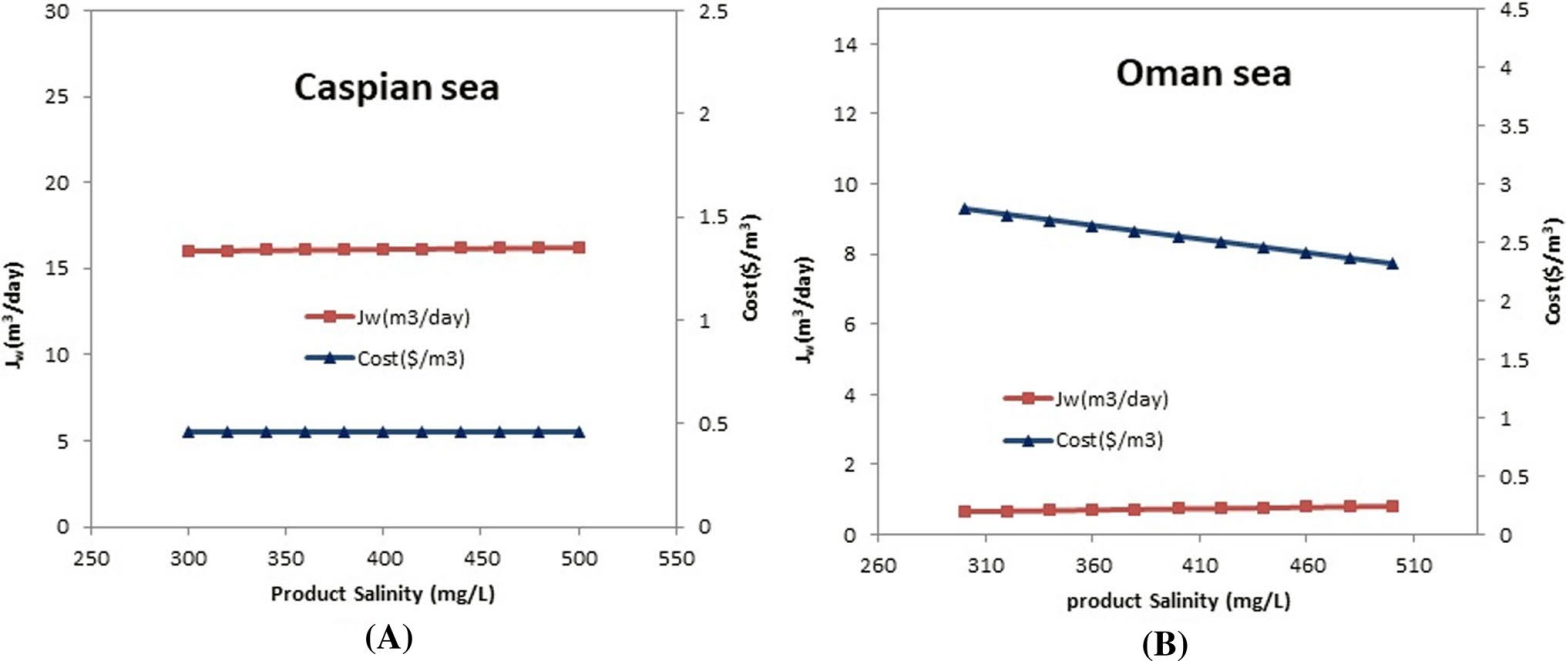
Evaluating the effect of the product water salinity on the cost and permeate flow rate (Δ*P* = 45 bar, *T* = 25 °C, *R*_Caspian_ = 0.55, *R*_Oman_ = 0.4, Feed salinity_Caspian_ = 17,000 mg/L, Feed salinity_Oman_ = 36,000 mg/L)

As can be seen in Fig. 7a and b, the effect of the product salinity on the cost and permeate flow rate is shown. Usually, the goal salinity of the product water depends on the field in which the water is used. The salinity ranges of the product water are selected from 300 to 500 ppm. As it is clear, there are not many changes in the Caspian Sea feed water case with the change of this parameter on the cost and permeate flow rate. However, in the case of Oman seawater, by decreasing in the salinity of the produced water to 500 ppm, there is a slight decrease in cost. In fact, it can be said that the proximity of feed and permeate water salinity is an important factor influencing the results of this parameter study.

### Evaluating the effect of temperature on the water cost and the permeate flow rate

**Fig.8:**
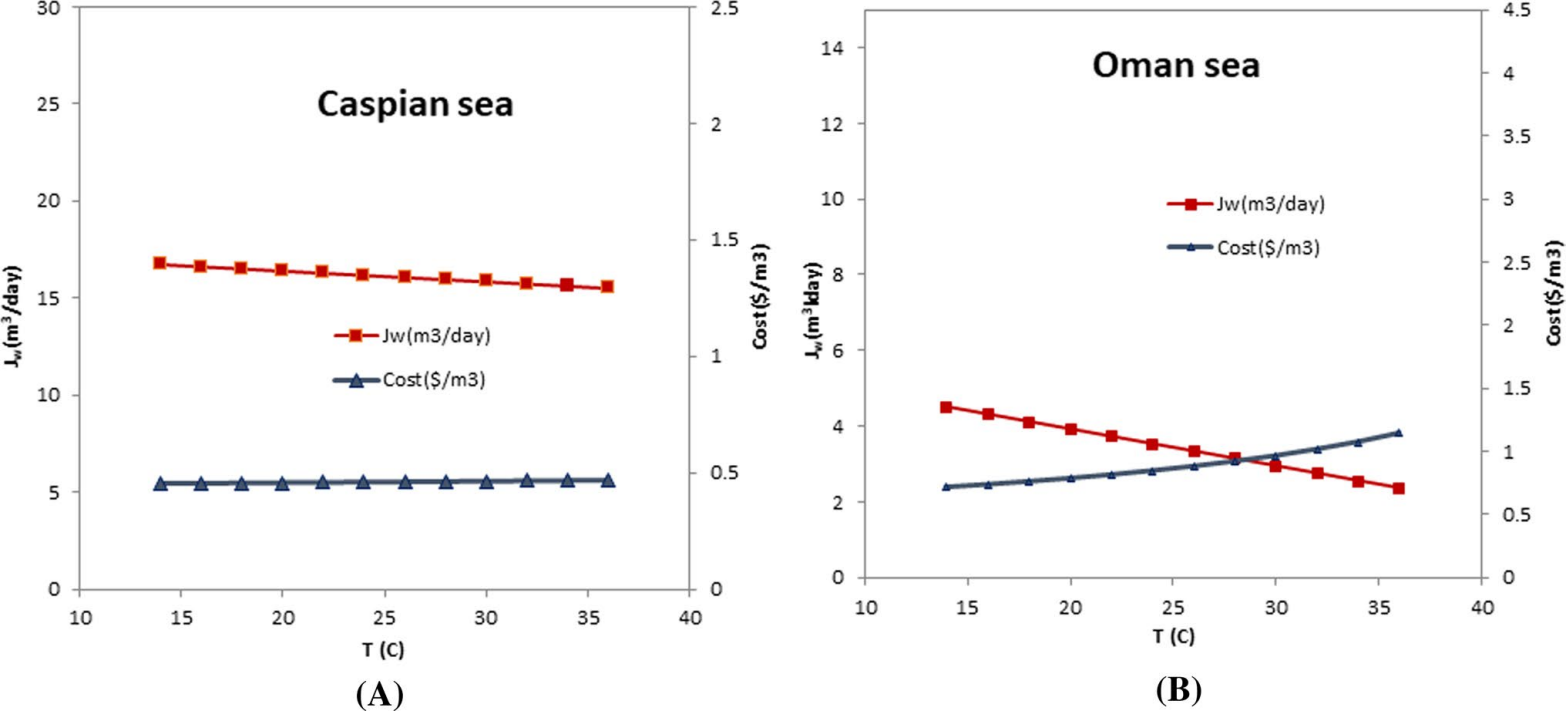
Evaluating the effect of the water temperature on the cost and the permeate flow rate (Δ*P* = 45 bar, *R*_Caspian_ = 0.55, *R*_Oman_ = 0.4, Feed salinity_Caspian_ = 17,000 mg/L, Feed salinity_Oman_ = 36,000 mg/L)

The last parameter used to model the Caspian, and Oman seawater desalination processes is the temperature, which is selected in the temperature range between 14 and 36 °C to study the changes in cost and the permeate flow rate. The results of this study present in Fig. 8a and b. It is clear that temperature changes have little effect on both of these parameters, and their relationship to temperature is inverse. However, it is easy to see that the temperature in the feed water of the Oman Sea is more effective in changing the cost and permeate flow rate of the membrane. In this case, as temperatures rise from 14 to 36 °C, the cost of product water increases from 0.712 to 1.141 $/m^3^. 3.6.

### Comparison of RO plant cost, for Caspian and Oman Sea intake seawater

Estimation of the costs for the two conceptual desalination plants with Caspian and Oman Sea as feed water, including the cost of the intake and pretreatment, cost of membrane elements replacement, the capital cost of the RO membrane, annual cost of the energy of the intake pump, cost of chemical treatment in the pretreatment, the annual cost of the power provided to the HPP, total annual O&M cost, is calculated separately and presented in Table 3. Comparing the costs of the RO desalination plant with the intake water of the Oman and Caspian Sea, the cost of the intake and pretreatment has the highest value among other costs, which is higher for the feed water of the Oman Sea than the water of the Caspian Sea. After that, the capital cost of the RO membrane is the highest cost for the RO plant for both feed water, but relatively this cost is higher for the Oman Sea. The reason for this difference is back to the difference in the membrane permeate flow rate. It is due to the difference in the salinity of the two water sources and, the difference in their osmotic pressure. Among the items calculated in terms of percentage difference, the capital cost of the RO membrane with a different percentage of 61.72% has the largest share of costs. After that, the cost of the intake and pretreatment at 20% and the cost of membrane elements replacement at 13.74% has significant share of costs. Under the same conditions for both feed water, the cost of product water from the Caspian Sea intake water is $/m^3^ 0.69, and for the Oman Sea, inlet water is $/m^3^ 1.24. These prices will change as each of the changes of the operating conditions. Due to the cost prices, it found that RO water desalination with Caspian Sea intake water costs about 50% less than Oman Sea feed water.

**Table 3:** Comparative Cost Analysis of conceptual RO desalination plant with capacity of 200,000 m^3^/day for two different intake water of Caspian and Oman sea (using membrane element DOW filmtec SW30HRLE)

Cost analysis elements $	Caspian sea	Oman sea	│Difference│	%Difference^b^
Cost of the intake and pretreatment	27,977,322	36,094,237	8,116,915	20.4
Cost of membrane elements replacement	1,021,176	6,488,341	5,467,165	13.74
Capital cost of the RO membrane	4,588,000	29,151,190	24,563,190	61.72
Annual cost of the energy of the intake pump (Iran)	737,073	1,013,445	276,372	0.69
Cost of chemical treatment in the pretreatment	6467	8892	2425	0.01
Annual cost of the power provided to the HPP	3,553,910	4,928,163	1,374,253	3.45
Total annual O&M cost	12,711,756	12,711,374	382	0.01
Summation of upper cost	50,595,704	90,395,642	39800702^a^	
Cost of produced water ($/m^3^)	1.24	0.69	0.55	

^a^Total difference

^b^This parameter is calculated rather than total difference

## Conclusions

In this work, by utilizing available economic theories of reverse osmosis (RO) desalination plants, the cost analysis of a conceptual plant with a production capacity of 200,000 m^3^/day was accomplished assuming the use of the Oman and Caspian seawater as feed. The effect of important parameters such as applied pressure, recovery, total salt content in feed, and product water and temperature has been studied theoretically. Due to the cost prices, it found that RO water desalination with Caspian Sea intake water costs about 50% less than Oman Sea feed water. It is due to the difference in the salinity of the two water sources and, the difference in their osmotic pressure. Despite this obvious result in the difference in the cost of water produced by reverse osmosis, it is necessary to examine other factors influencing the implementation of such a project, including determining the amount of water that can be harvested, environmental assessment, estimating water transfer requirements, and so on.

## List of symbols

*A*: Area (m^2^)
*A*_*w*_: Permeability coefficient (m/s-Pa)
*B*_*s*_: Solute transport parameter (m/s)
*C*: Average salinity through the membrane element (mol/m^3^)
*C*_*ch*_: Cost of chemical treatment ($/m^3^)
CD_*m*_: Membrane cost ($)
*C*_*e*_: Unit power cost ($/kWh)
*C*_*f*_: Concentration of feed water (mol/m^3^)
*C*_*m*_: Solute concentration in the membrane (mol/m^3^)
*C*_RO_: Mass fraction of salt in permeate (%)
*C*_*p*_: Solute concentration at permeate (mol/m^3^)
*C*_*r*_: Concentration in the concentrate (mol/m^3^)
*C*_*w*_: Water concentration in the membrane (mol/m^3^)
*F*_1_: Plant load factor, %
*i*_*eff*_: Effective discount rate relation between the future value and present value
*J*_*s*_: Solute transport (m/s)
*J*_*w*_: Permeate flux (m/s)
*N*: Number of membrane elements
PC_m_: Cost per membrane ($)
*P*_*f*_: Feed water pressure (Pa)
*P*_IP_: Pressure after the intake pump (bar)
*P*_*m*_: Annual membrane replacement factor (%)
*P*_*p*_: Permeate pressure (Pa)
Δ*P*: Transmembrane pressure difference (Pa)
*Q*_*f*_: Feed flow rate (m^3^/day)
$$\dot{Q}_{f}$$: Daily feed flowrate after extracting the bypass ratio (m^3^/day)
*Q*_*p*_: Permeate flow rate (m^3^/day)
$$\dot{Q}_{{{\text{P}} \cdot {\text{a}}}}$$: Annual volume flow rate of product water (m^3^)
*Q*_*p*__, el_: Permeate flow rate per membrane element (m^3^/s)
$$\dot{Q}_{P}$$: Mass flow rate of permeate in one element (kg/s)
*Q*_*r*_: Rejected flow rate (m^3^/day)
*R*: Gas constant (J/mol-k)
*r*_*n*_: Nominal escalation rate which effects of resource depletion, increased demand and inflation (%)
*r*_*r*_: Recovery ratio (%)
*R*_*s*_: Salt rejection (%)
*T*: Temperature (K)
TCF: Temperature correction factor at *T* (%)
*V*_*w*_: Water molar volume (m^3^)

## Greek symbols

*η*: Efficiency (%)
Δ*π*: Osmotic pressure difference (Pa)

## Subscripts

*E*: Energy
*f*: Feed water
IP: Intake Pump
*m*: Membrane

## References

[CR1] Al-Obaidi MA, Filippini G, Manenti F, Mujtaba IM (2019). Cost evaluation and optimisation of hybrid multi effect distillation and reverse osmosis system for seawater desalination. Desalination.

[CR2] Daneshmand H, Mahmoudi P (2017). Estimation and assessment of temporal stability of periodicities of droughts in Iran. Water Resour Manag.

[CR3] Debele B (2019) The role of desalination in an increasingly water-scarce world, international bank for reconstruction and development, The World Bank,1818 H Street NW, Washington, DC 20433

[CR4] El-Emam RS, Dincer I (2014). Thermodynamic and thermoeconomic analyses of seawater reverse osmosis desalination plant with energy recovery. Energy.

[CR5] Elimelech M, Phillip WA (2011). The future of seawater desalination: energy, technology, and the environment. Science.

[CR6] Emamjome A, Zahedi MM, Ziyaadini M (2019). Economic analysis for process optimization of Chabahar Maritime University reverse osmosis desalination plant: a case study. Appl Water Sci.

[CR7] Farhoudi G, Poll K (1992). A morphotectonic study of environmental impact on ground water in Southern Iran and under the Persian Gulf. Geol Rundsch.

[CR8] La Cerva M, Gurreri L, Cipollina A, Tamburini A, Ciofalo M, Micale G (2019). Modelling and cost analysis of hybrid systems for seawater desalination: Electromembrane pre-treatments for Reverse Osmosis. Desalination.

[CR9] Lee KP, Arnot TC, Mattia D (2011). A review of reverse osmosis membrane materials for desalination—development to date and future potential. J Membr Sci.

[CR10] Malek A, Hawlader MNA, Ho JC (1996). Design and economics of RO seawater desalination. Desalination.

[CR11] Marcovecchio MG, Aguirre PA, Scenna NJ (2005). Global optimal design of reverse osmosis networks for seawater desalination: modeling and algorithm. Desalination.

[CR12] Oki T, Kanae S (2006). Global hydrological cycles and world water resources. Science.

[CR13] Pourmortazavi SM, Taghdiri M, Ahmadi R, Zahedi MM (2017). Procedure optimization for removal of 2,4-dichlorophenoxyacetic acid from water by surfactant-modified magnetic nanoparticles. Desalt Water Treat.

[CR14] Sarai Atab M, Smallbone AJ, Roskilly AP (2016). An operational and economic study of a reverse osmosis desalination system for potable water and land irrigation. Desalination.

[CR15] Van der Bruggen B, Vandecasteele C (2002). Distillation vs. membrane filtration: overview of process evolutions in seawater desalination. Desalination.

[CR16] Voutchkov N (2013). Desalination engineering planning and design.

[CR17] Voutchkov N (2018). Energy use for membrane seawater desalination—current status and trends. Desalination.

[CR18] Wenten IG, Khoiruddin (2016). Reverse osmosis applications: prospect and challenges. Desalination.

[CR19] Wilf M, Schierach MK (2001). Improved performance and cost reduction of RO seawater systems using UF pretreatment. Desalination.

[CR20] Yang Z, Ma XH, Tang CY (2018). Recent development of novel membranes for desalination. Desalination.

[CR21] Zahedi MM, Ghasemi SM (2017). Separation study of Mg+2 from seawater and RO brine through a facilitated bulk liquid membrane transport using 18-Crown-6. J Water Reuse Desalin.

